# Boosting *Escherichia coli*’s heterologous production rate of ectoines by exploiting the non-halophilic gene cluster from *Acidiphilium cryptum*

**DOI:** 10.1007/s00792-020-01188-8

**Published:** 2020-07-22

**Authors:** Lukas Bethlehem, Katharina D. Moritz

**Affiliations:** Institute for Microbiology and Biotechnology, University Bonn, Meckenheimer Allee 168, 53115 Bonn, Germany

**Keywords:** Compatible solutes, Hydroxyectoine, Ectoine, Industrial applications, *Escherichia coli*, *Acidiphilium cryptum*

## Abstract

The compatible solutes ectoine and hydroxyectoine are synthesized by many microorganisms as potent osmostress and desiccation protectants. Besides their successful implementation into various skincare products, they are of increasing biotechnological interest due to new applications in the healthcare sector. To meet this growing demand, efficient heterologous overproduction solutions for ectoines need to be found. This study is the first report on the utilization of the non-halophilic biosynthesis enzymes from *Acidiphilium cryptum* DSM 2389^T^ for efficient heterologous production of ectoines in *Escherichia coli*. When grown at low salt conditions (≤ 0.5% NaCl) and utilizing the cheap carbon source glycerol, the production was characterized by the highest specific production of ectoine [2.9 g/g dry cell weight (dcw)] and hydroxyectoine (2.2 g/g dcw) reported so far and occurred at rapid specific production rates of up to 345 mg/(g dcw × h). This efficiency in production was related to an unprecedented carbon source conversion rate of approx. 60% of the theoretical maximum. These findings confirm the unique potential of the here implemented non-halophilic enzymes for ectoine production processes in *E. coli* and demonstrate the first efficient heterologous solution for hydroxyectoine production, as well as an extraordinary efficient low-salt ectoine production.

**Electronic supplementary material:**

The online version of this article (10.1007/s00792-020-01188-8) contains supplementary material, which is available to authorized users.

## Introduction

Compatible solutes are small organic compounds, which are accumulated or synthesized by microorganisms under physical stress conditions enabling protection of whole cells and macromolecules against, for example, high osmolarity, heat, freezing, and desiccation (Manzanera et al. 2004; Borges et al. 2002; Göller and Galinski 1999; Kempf and Bremer 1998; Louis et al. 1994; Lippert and Galinski 1992). For this purpose, they can be enriched up to molar concentrations in a cell without adverse effects on its metabolism (Brown 1976). Ectoine and hydroxyectoine are derivatives of the amino acid aspartate and are widely abundant osmo- and heat-protectants in halophilic microorganisms. Both compounds share potent moisturizing and skin protective features, which led to implementations of especially ectoine into a multitude of skincare and well-being products (Pastor et al. 2010). Compared to ectoine, hydroxyectoine displays even higher water-binding activities and enhanced glass-forming attributes, making this compatible solute a potent desiccation protectant (Manzanera et al. 2004; Tanne et al. 2014). Furthermore, ectoine and hydroxyectoine are distinct in their effect on DNA. While ectoine lowers the melting temperature of double-stranded DNA, hydroxyectoine conversely elevates it (Seip et al. 2011). Recently, the field of application is expanding due to studies demonstrating anti-inflammatory effects of ectoine and ectoine derivatives in intestinal bowel disease and carbon nanoparticle induced airway inflammation, as well as protein stabilizing effects in neurodegenerative diseases (Abdel-Aziz et al. 2013, 2015; Bazazzadegan et al. 2017; Unfried et al. 2014). Promising applications as adjuvant treatment in these diseases led to a gain in CAGR (calculated annual growth rate) for the ectoine market and therefore to a demand for feasible biotechnological production processes (Becker and Wittmann 2020).

Ectoine is currently produced and marketed at large scale primarily utilizing the natural producer strain *Halomonas elongata* and optimized strain derivatives of this organism (Kunte et al. 2014). In contrast to ectoine, hydroxyectoine production in *H. elongata* is only possible under substantial accumulation of its precursor ectoine (Wohlfarth et al. 1990). As a consequence, purification of hydroxyectoine would require a time- and cost-intensive chromatographic separation of both solutes, thus restricting relevant current applications to ectoine only. A viable hydroxyectoine production process would allow to screen for new applications with this compound due to the distinctive features of hydroxyectoine. However, also the production of ectoine in *H. elongata* is a cost-intensive process, as it demands high concentrations of sodium chloride (NaCl), which makes expensive desalting and salt disposal during downstream processing a necessary requirement.

To meet the demand for more efficient production of ectoines (= ectoine + hydroxyectoine), increasing numbers of heterologous production strains have been generated in recent years that enable an extracellular accumulation of ectoines at low NaCl concentrations (Parwata et al. 2019; Gießelmann et al. 2019; Czech et al. 2018; Pérez-García et al. 2017; Ning et al. 2016; He et al. 2015; Eilert et al. 2013; Becker et al. 2013; Schubert et al. 2007). Interestingly, the employed biosynthesis enzymes for heterologous production of ectoines originated from halophilic donors with optimum activities in the presence of salts. This might be a reasonable approach for ectoine production in the Gram-positive host *Corynebacterium glutamicum*, which naturally exhibits a relatively high ionic strength in its cytoplasm (Krämer et al. 1990; Follmann et al. 2009). In the Gram-negative host *Escherichia coli*, however, activities of halophilic enzymes could be significantly impaired under low salt conditions, due to the lower ionic strength in its cytoplasm (Dinnbier et al. 1988). In this study, we therefore used the biosynthesis gene cluster from the non-halophilic organism *Acidiphilium cryptum* for the heterologous production of ectoines in *E. coli*.

**Fig. 1:**
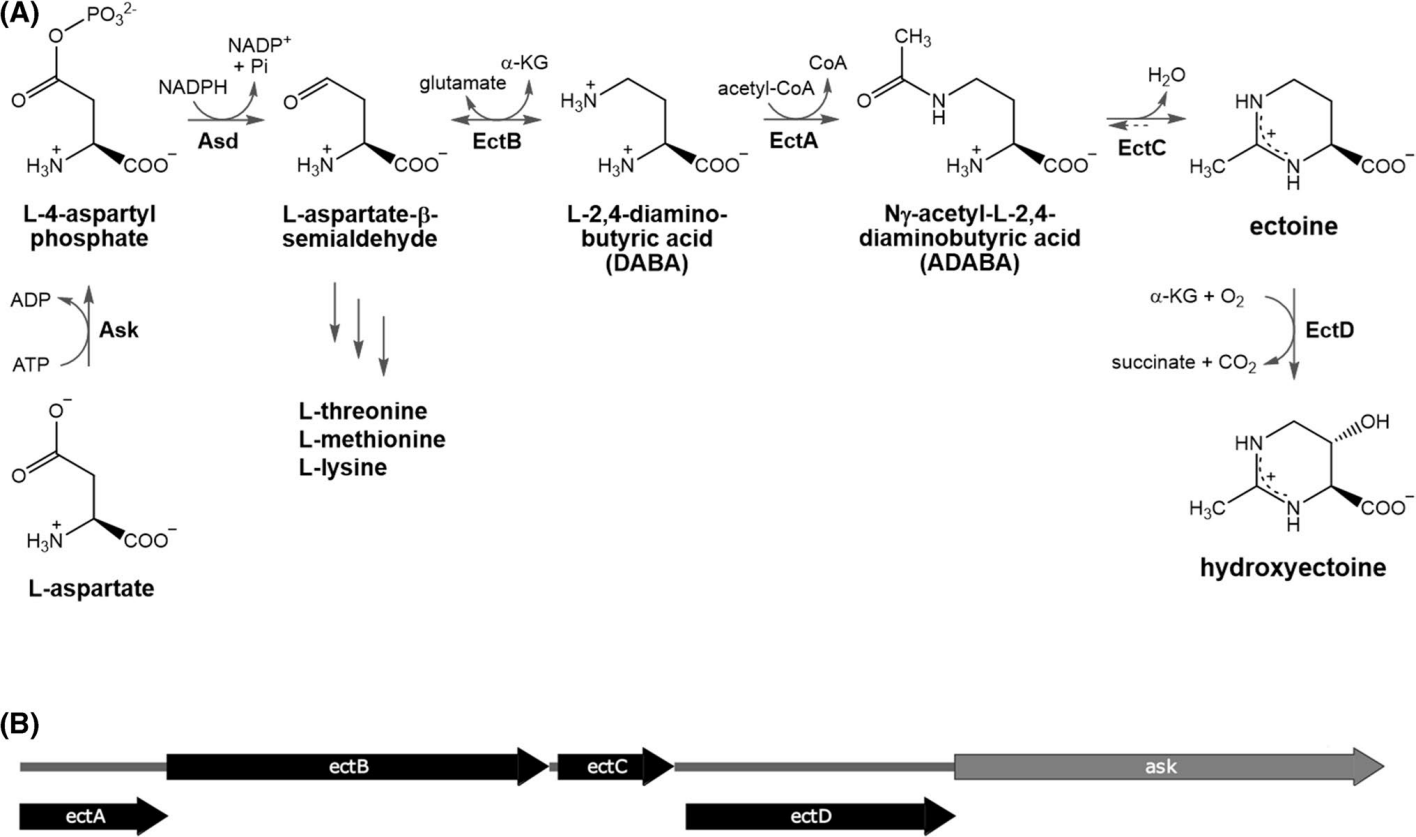
Pathway of ectoine and hydroxyectoine biosynthesis. **a** Biosynthesis of ectoine and hydroxyectoine starts with the amino acid L-aspartate. L-aspartate-β-semialdehyde describes a joint precursor for the aspartate amino acid family and ectoines. *Ask* aspartokinase, *Asd* aspartate-β-semialdehyde dehydrogenase, *EctB* L-2,4-diaminobutyric acid transaminase, *EctA* L-2,4-diaminobutyric acid acetyltransferase, *EctC* ectoine synthase, *EctD* ectoine hydroxylase, *α-KG* α-ketoglutarate, *CoA* Coenzyme A. **b** The *A. cryptum* hydroxyectoine biosynthesis gene cluster comprises the genes for acetyltransferase (*ectA*), transaminase (*ectB*), ectoine synthase (*ectC*), ectoine hydroxylase (*ectD*), as well as an additional aspartokinase gene (*ask*) overlapping ectD

The biosynthetic pathway of ectoine and hydroxyectoine is well-studied (Peters et al. 1990; Bursy et al. 2007; Czech et al. 2019) and branches from the biosynthesis of the aspartate amino acid family (Fig. 1a). The transaminase EctB catalyses the transamination of the precursor l-aspartate-β-semialdehyde to l-2,4-diaminobutyric acid (DABA). The following acetylation to Nγ-acetyl-l-2,4-diaminobutyric acid (ADABA) is carried out by the acetyltransferase EctA, followed by the cyclization to ectoine catalysed by the ectoine synthase EctC. For the conversion to hydroxyectoine, the ectoine ring is hydroxylated by the ectoine hydroxylase EctD. Usually, these biosynthetic genes are organized in one operon, which is in some organisms extended by an *ask* gene coding for an additional aspartokinase. This enzyme catalyses the second step in the pathway of the aspartate amino acid family, phosphorylating l-aspartate to l-4-aspartyl phosphate (Fig. 1a). This step can be a critical bottleneck for the ectoine biosynthesis, due to a possible feedback inhibition of the Ask protein by the end products of the aspartate amino acid family (threonine, methionine, lysine) (Bestvater et al. 2008). Such an additional *ask* gene is also present in the here used hydroxyectoine biosynthesis gene cluster of *A. cryptum* DSM 2389^T^ overlapping the *ectD* gene (Fig. 1b).

*A. cryptum* is an acidophilic α-proteobacterium which thrives in acidic, metal-rich environments (Harrison 1981). It was shown that *A. cryptum* is capable of producing the compatible solute hydroxyectoine in response to elevated NaCl or Al_2_(SO_4_)_3_ levels, despite the organism’s rather weak salt tolerance of up to 5% NaCl (Moritz et al. 2015). The *A. cryptum* hydroxyectoine biosynthesis proteins differ from halophilic variants by their less acidic nature implying optimum activity in the absence of salt, as experimentally shown for the EctC enzyme activity (Moritz et al. 2015). We therefore hypothesized, that this capacity could enable more efficient overproduction of ectoines at low salt conditions in non-halophilic host organisms with a low ionic strength in their cytoplasm. In this study, the productivity of the unique biosynthesis proteins of *A. cryptum* was tested for the first time under optimized conditions in *E. coli* in order to prove their potential for the heterologous hydroxyectoine and ectoine production under low salt conditions.

## Materials and methods

### Bacterial strains, media, and cultivation

All strains in this study were acquired from the DSMZ (Braunschweig, Germany). The type strain *A. cryptum* DSM 2389^T^ (Harrison 1981) was used as the donor for the hydroxyectoine biosynthesis gene cluster. Cultivation was performed in modified M269 medium (Harrison 1981), containing (in g/L): (NH_4_)_2_SO_4_ (2.0), KCl (0.1), K_2_HPO_4_ (0.5), MgSO_4_ × 7 H_2_O (0.5), yeast extract (0.3), glycerol (5.0). The strain *E. coli* DH5α DSM 6897 (Hanahan 1983) (F^−^ φ80*lacZ*ΔM15 Δ(*lacZYA-argF*)U169 *deoR recA*1 *endA*1 *hsdR*17(rk^−^ mk^+^) *phoA supE*44 λ^−^ *thi*-1 *gyrA*96 *relA*1) was used as the host for transformation experiments as well as for hydroxyectoine and ectoine overproduction experiments. For cloning steps, *E. coli* DH5α was cultivated in LB medium (Bertani 1951). For overproduction of ectoines the defined medium MM63 was utilized (Larsen et al. 1987), containing (in g/L): KH_2_PO_4_ (13.61), KOH (4.21), (NH_4_)_2_SO_4_ (1.98), MgSO_4_ × 7 H_2_O (0.25), FeSO_4_ × 7 H_2_O (0.0011), and glucose-monohydrate (5.0) or glycerol (4.65). NaCl was added to the medium in concentrations of 0–1% and is stated if relevant. A vitamin solution was added to enable growth of *E. coli* DH5α (Imhoff and Trüper 1977), resulting in final concentrations (in g/L): nicotinamide (0.35), Ca-DL-pantothenate (0.10), thiamine × HCl (0.30), *p*-aminobenzoic acid (0.20), pyridoxal chloride (0.10), cyanocobalamin (0.05), biotin (0.10). For plasmid maintenance, 100 mg/L of the antibiotic carbenicillin were added. All cultivations were performed aerobically at 37 °C and 180 rpm in 250 mL shake flasks with baffles.

### Construction of plasmids

For the heterologous production of ectoines in *E. coli* utilizing the hydroxyectoine biosynthesis gene cluster from *A. cryptum*, plasmids based on the pASK-IBA3 vector (IBA Lifesciences, Göttingen, Germany) were constructed. The vector comprises a *bla* gene, a ColE1 replicon, and a *tet* promoter, which enables high expression levels after induction with anhydrotetracycline (AHT). Plasmid constructions were performed by standard restriction cloning, and plasmids were integrated into chemically competent *E. coli* DH5α cells via transformation. The sequencing of plasmids was carried out by Eurofins Genomics (Ebersberg, Germany). Two different plasmids were designed, containing either *ectABCDask* or *ectABCD*, allowing to compare hydroxyectoine production with and without the additional aspartokinase from *A. cryptum* (Table 1; Figure S1, Supplementary material). The plasmid pASK_ectABCDask was constructed in a two-step cloning process using the PstI restriction site present in *ectD* to ligate both fragments. Construction of pASK_ectABCD was performed in one-step cloning. The plasmid pASK_ectABCD_m_ was found to result in ectoine production due to a random point mutation in *ectD* and was used for most ectoine overproduction processes in this study. The plasmid pASK_ectABC was constructed in a one-step restriction cloning after detection of the random mutagenesis to exclude any detrimental effects of *ectD*_m_ on ectoine production (Table 1). The plasmid pASK_ectAB-RectC was designed in a multiple cloning process to assess the effect of *ectC* overexpression on ADABA accumulation during ectoine overproduction (Table 1; Figure S2, Supplementary material). In this case, the expression of *ectC* is under the control of a separate *tet* promoter and the optimized ribosome binding site (RBS) from the pET-22b(+) vector.

**Table 1:** Information on plasmid constructions

Construct		Label	Nucleotide sequence (5′–3′)	Restriction site
ectABCDask	Step 1	fwd1	ATTTCTAGACGGTGGCGCTGCGTC	XbaI
		rev1	CCGACTGCAGCAGCGCGATCT	PstI
	Step 2	fwd2	AGATCGCGCTGCTGCAGTCG	PstI
		rev2	ATTAAGCTTCGCCCGCATGCACGA	HindIII
ectABCD,		fwd1		
ectABCD_m_		rev3	ATTAAGCTTTCGGAGCCATGCTCATGC	HindIII
ectABC		fwd1		
		rev4	AATAAGCTTCGTCAGGCCGCCTCGCCGA	HindIII
ectAB-RectC	Step 1	fwd3	GTGCATATGATCATCCGGACTCTGAAGGAG	NdeI
		rev5	ATTCTCGAGGGCCGCCTCGCCGACC	XhoI
	Step 2	fwd4	TAATACGACTCACTATAGG	
		rev6	ATTAAGCTTGCGGTGGCAGCAGCCAACT	HindIII
	Step 3	fwd5	ATTCCATGGTGGCCAGATGATTAATTCCT	NcoI
		rev7	ATTAAGCTTTCAGGCCGCCTCGCCGA	HindIII
	Step 4	fwd1		
		rev8	ATTCCATGGCAGAGTCCGGATGATCATG	NcoI

Primers used for restriction cloning of hydroxyectoine (ectABCDask, ectABCD) and ectoine (ectABCD_m_, ectABC, ectAB-RectC) overproduction plasmids are listed. Restriction sites are underlined

### Overproduction, detection, and quantification of ectoines

Precultures of the production strains were prepared in the same medium as the main cultures and incubated overnight at 37 °C and 180 rpm. The preculture was centrifuged at 4500×*g* and cells were resuspended in fresh medium. Main cultures (100 mL MM63) were inoculated to an optical density at 600 nm (OD_600_) of 0.1 and induced at OD_600_ of 0.3–0.4 by adding 0.2 mg/L AHT. Cells were harvested in the stationary phase and freeze-dried by lyophilization for calculation of the dry cell weight (dcw). To determine the intracellular solute concentration, the freeze-dried cells were extracted using a modified Bligh and Dyer protocol (1959), as described previously (Galinski and Oren 1991). The water-soluble fraction and the culture supernatant were analysed by isocratic high-performance liquid chromatography (HPLC) using a LiChrospher amino phase column (Merck, Darmstadt, Germany) and acetonitrile/water (80/20% v/v) as the mobile phase. Measurements were carried out at ambient temperature and a flow rate of 1 mL/min. For quantification of ectoine and hydroxyectoine, UV and refractive index detection were employed as described by Galinski and Herzog (1990). The overproduction experiments were carried out in at least triplicates (*n* = 3–20) and the results displayed with standard deviation. The specific production rate over time is given as mean with upper/lower boundary (*n* = 2).

### Detection and quantification of by-products and carbon sources

For detection of zwitterionic and uncharged by-products (e.g. trehalose), the culture supernatant was analysed by isocratic HPLC as described for detection of ectoines. Quantification of glycerol, glucose, and acetate in the culture medium was performed by isocratic HPLC with an Aminex HPX-87H column (Bio-Rad, Hercules, USA). As a mobile phase, 0.02 N H_2_SO_4_ was used at a flow rate of 0.6 mL/min. For eluent detection, UV and refractive index detection were employed. Furthermore, N-reactive compounds in the culture supernatant were analysed by gradient HPLC with pre-column FMOC-ADAM-derivatization as described previously (Kunte et al. 1993). The concentration of the precursor ADABA was determined by this method.

### Carbon balance calculation

The carbon balance calculation was carried out for the production strains based on overproduction experiments under optimum growth conditions, with the carbon source entirely consumed and no other products than ectoines detected in the culture medium. The control strain *E. coli* DH5α with the empty vector (pASK-IBA3), which generates only biomass under the same conditions, was used to elucidate the carbon input for the heterologous production of ectoines separately from the biomass formation. In the case of hydroxyectoine production, the ectoine share of 3.6% was not considered when calculating the carbon balance.

### Protein expression and separation via SDS-PAGE

A protein expression experiment was performed to visualize the production of the heterologous proteins in the constructed *E. coli* strains. The cells were cultivated as described before and protein expression was induced by the addition of AHT (0.4 mg/L). Before and after induction, 10 mL samples were taken for protein analysis. The cell pellets were processed in a standard protein extraction with lysozyme and sodium dodecyl sulfate (SDS). After separating the soluble protein fraction by centrifugation at 4 °C and 16,000×*g* for 30 min, its protein content was determined by the bicinchoninic acid assay after Smith et al. (1985). The soluble protein extracts were analysed semi-quantitative by SDS polyacrylamide gel electrophoresis (SDS-PAGE) after Laemmli (1970). In the case of *E. coli* DH5α pASK_ectABCD_m_ and pASK-IBA3 20 µg of soluble protein extracts were loaded on the gel, and in the case of *E. coli* DH5α pASK_ectAB-RectC 10 µg of soluble proteins were applied.

## Results

### Hydroxyectoine production

**Fig. 2:**
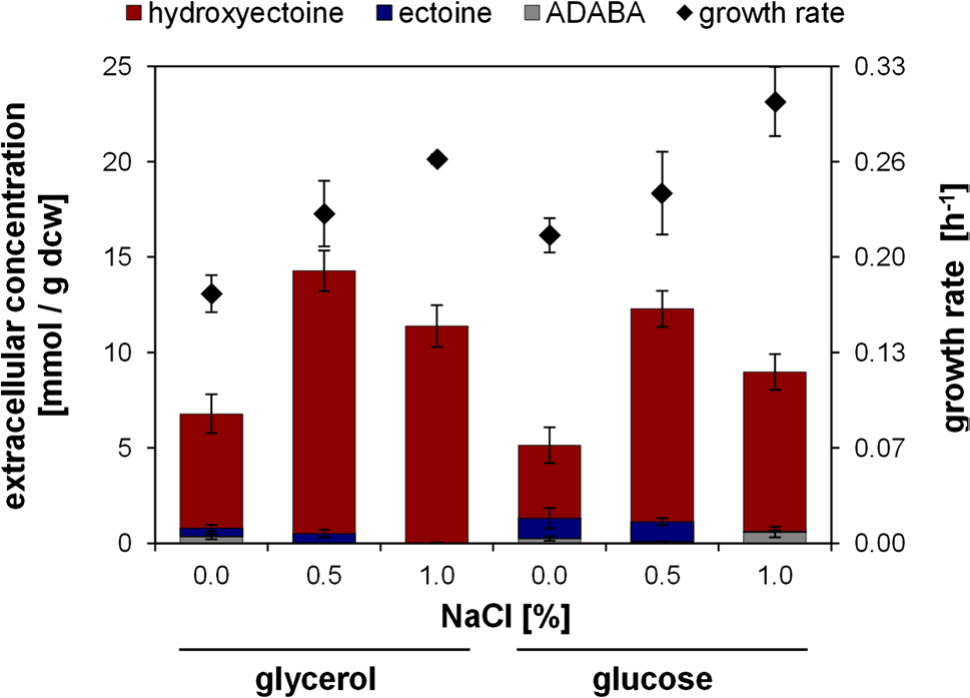
Hydroxyectoine production in *E. coli* DH5α pASK_ectABCDask. The production strain was cultured in MM63 with 0–1% NaCl and 50 mM glycerol or 25 mM glucose as carbon source. The specific extracellular content of hydroxyectoine and its precursors (ADABA, ectoine) in mmol/g dry cell weight (dcw), as well as the growth rate (h^–1^), were determined. All experiments were performed at least in triplicates. Error bars indicate the standard deviation (n = 3–9)

For hydroxyectoine production the strain *E. coli* DH5α pASK_ectABCDask was used, expressing the entire hydroxyectoine biosynthesis gene cluster from *A. cryptum* under control of the AHT-inducible *tet* promoter. For overproduction experiments, the strain was cultivated in shake flasks utilizing minimal medium with a low NaCl concentration (0–1%) and either 25 mM glucose or 50 mM glycerol as carbon source. The growth rate of cultures increased for both carbon sources with the addition of NaCl and was slightly higher for glucose cultures, compared to glycerol cultures. Under all conditions, high concentrations of hydroxyectoine were produced and naturally excreted into the cultivation medium, with less than 1% of the total ectoines remaining within the cells (Fig. 2). Final product concentrations in the medium ranged from 4.2 to 8.3 mM in glucose cultures and from 5.9 to 10.0 mM in glycerol cultures, depending on the NaCl content. Thus, glycerol-grown cultures exhibited higher extracellular hydroxyectoine concentrations in all experiments. Besides hydroxyectoine, the precursors ADABA (< 6.5%) and ectoine (< 20%) were detected in the medium but no other relevant N-reactive compounds (other than ADABA) nor side products like trehalose were detectable by the HPLC methods employed. The highest specific extracellular hydroxyectoine content of 13.8 mmol/g dcw (2.2 g/g dcw) with a low precursor share of 3.6% was achieved utilizing glycerol medium and 0.5% NaCl (Fig. 2). These conditions were the best tested for heterologous hydroxyectoine production in this study.

We then asked whether the presence of the *ask* gene in the gene cluster had a positive effect on overall hydroxyectoine production. For this purpose, we constructed another production strain, *E. coli* DH5α pASK_ectABCD, excluding the additional aspartokinase from *A. cryptum*. However, this strain showed no significant difference in hydroxyectoine production compared to the original production strain (*E. coli* DH5α pASK_ectABCDask) (Figure S3, Supplementary material). Thus, the presence of the *ask* gene from *A. cryptum* had only a marginally beneficial effect on hydroxyectoine production under the conditions employed.

### Ectoine production

During strain construction for hydroxyectoine overproduction, one strain was found to have a random point mutation in *ectD*. The mutation resulted from the substitution of leucine to proline at position 118 in the EctD protein. Experiments showed that this strain, called *E. coli* DH5α pASK_ectABCD_m_, was not able to produce hydroxyectoine, but accumulates ectoine in high amounts (≥ 7 mM) (Figure S3, Supplementary material). This strain was therefore used for ectoine overproduction experiments in this study. Later, it was confirmed that *E. coli* DH5α expressing only the genes *ectABC* revealed no significant difference in ectoine production and growth (data not shown).

**Fig. 3:**
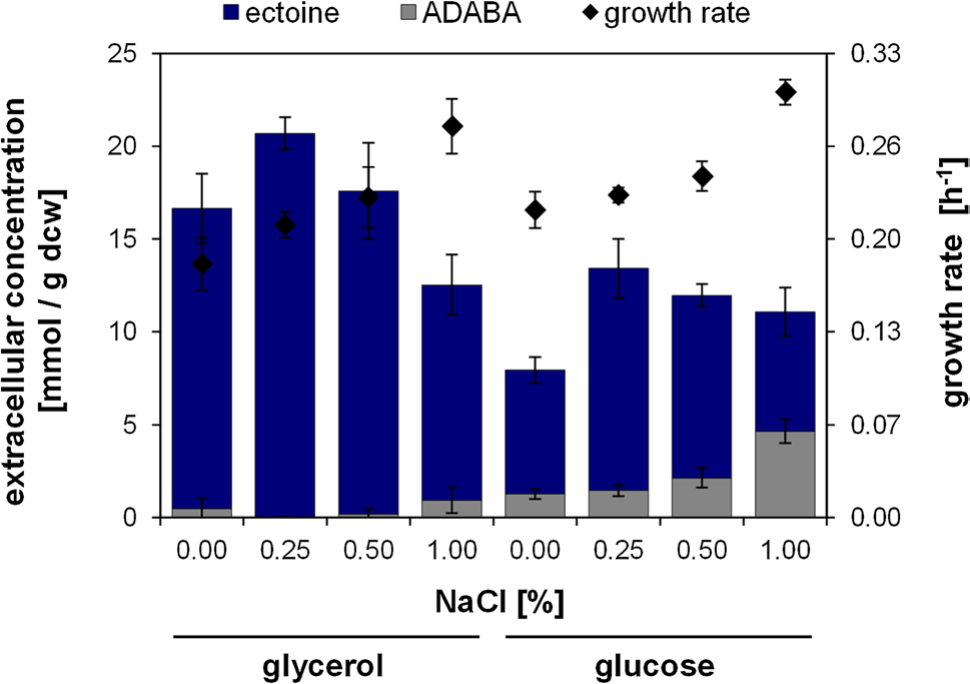
Ectoine production in *E. coli* DH5α pASK_ectABCD_m_. The production strain was cultured in MM63 with 0–1% NaCl and 50 mM glycerol or 25 mM glucose as carbon source. The specific extracellular content of ectoine and its precursor ADABA in mmol/g dry cell weight (dcw), as well as the growth rate (h^–1^), were determined. All experiments were performed at least in triplicates. Error bars indicate the standard deviation (n = 3–20)

Ectoine production was performed under the same conditions as described for hydroxyectoine production, except for an additional NaCl concentration (0.25%) in order to further specify the process optimum (Fig. 3). Again, the growth rates were higher for the glucose cultures and increased with increasing NaCl concentrations. As with hydroxyectoine, high concentrations of ectoine were naturally excreted into the culture medium, and only small amounts (≤ 1%) were detected within the cells under optimized conditions. Ectoine concentrations in the medium ranged from 5.5 to 9.2 mM in glucose cultures and from 8.7 to 11.7 mM in glycerol cultures. Furthermore, only small amounts of the precursor ADABA were detected in glycerol-grown cultures (0–7%), whereas in glucose-grown cultures the extracellular ADABA concentration was significantly higher (11–42%). Again, relevant N-reactive compounds other than ADABA, or side products like trehalose, were not detected by the HPLC methods employed. The remarkable maximum specific extracellular ectoine content of 20.6 mmol/g dcw (2.9 g/g dcw) was achieved in medium with glycerol and 0.25% NaCl (Fig. 3). Under these conditions, no precursor or by-products were detected in the supernatant. Similar to hydroxyectoine production a low NaCl concentration and glycerol as carbon source proofed to be optimal for the heterologous production of ectoine in this study.

### Specific production rate of ectoines over time

**Fig. 4:**
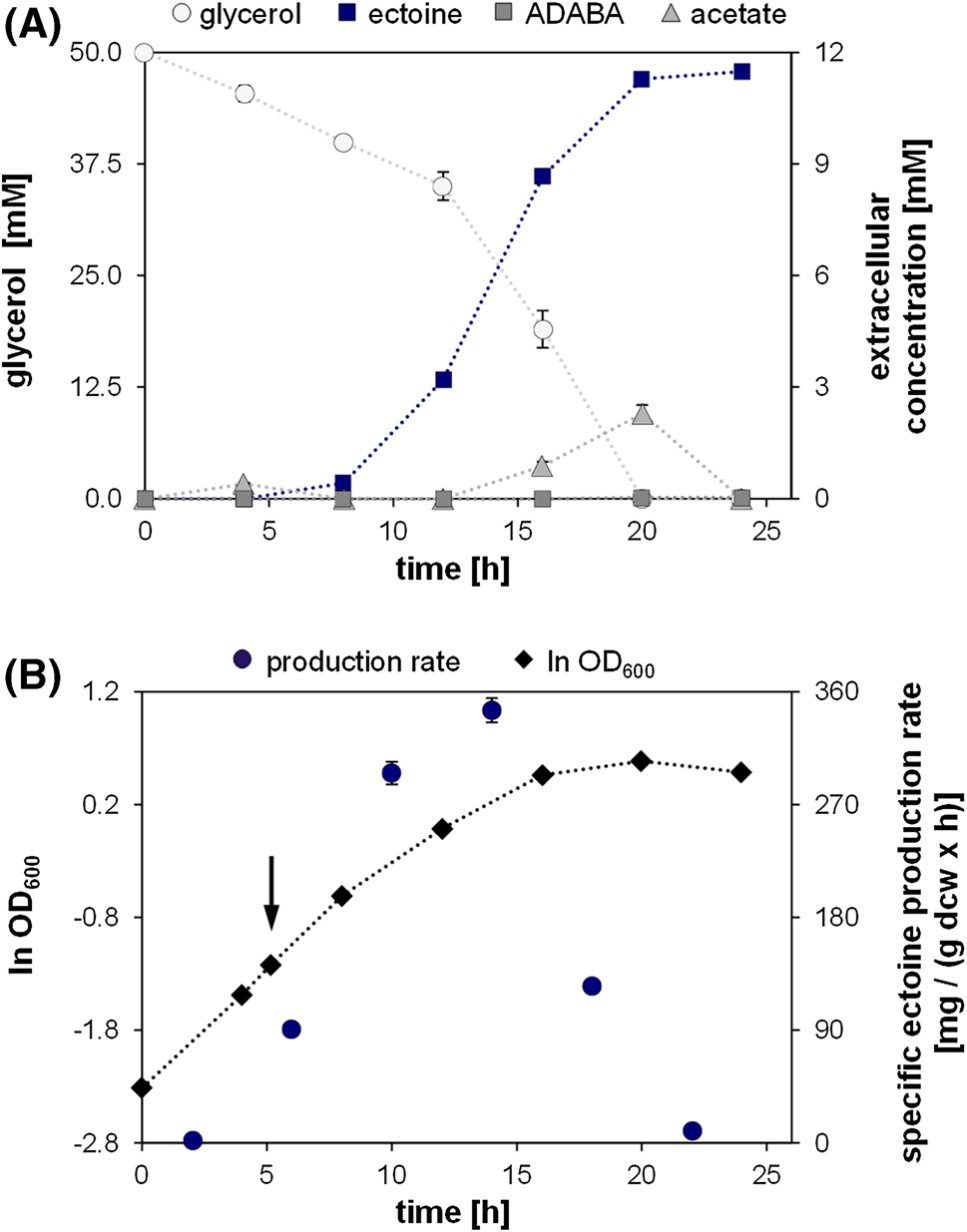
Ectoine production process over time in *E. coli* DH5α pASK_ectABCD_m_. The production strain was cultured in MM63 supplemented with 0.25% NaCl and 50 mM glycerol. **a** Extracellular accumulation of ectoine and side products, as well as glycerol depletion, were determined over time. **b** The natural logarithm of the growth curve as obtained from the optical density at 600 nm (ln OD_600_) is displayed and the time point of induction is indicated by an arrow. The time course of specific ectoine production rate in mg/(g dcw × h) was approximated by calculating the difference in ectoine concentration at two sampling points divided by the time difference and the mean dry cell weight (dcw). Shown are mean values of two experimental replicates

To characterize the production process of ectoine in our strains in more detail, the specific production rates for *E. coli* DH5α pASK_ectABCD_m_ were determined. Therefore, samples were taken in 4-h intervals during the ectoine overproduction in shake flask experiments under optimal conditions (MM63 with 0.25% NaCl and glycerol). The process was monitored for concentrations of the substrate glycerol and the main product ectoine, as well as for concentrations of the precursor ADABA and the side product acetate (Fig. 4a). After induction, ectoine formation and accumulation in the culture medium started and increased rapidly for the next 10 h, until glycerol was depleted. The side product acetate was excreted in moderate amounts (2.3 mM) after 12 h of the process and was consumed after the main carbon source was no longer available (20–24 h). The precursor ADABA was not detected during the entire process. This confirms previous experiments showing that ADABA is, if at all, only formed in low amounts (< 7%) when glycerol is used as carbon source. From these data, the time-course of the specific production rate in mg/(g dcw × h) could be determined, which reached peak values at the late exponential growth phase (Fig. 4b). The maximum specific production rate for the ectoine production process was 345 mg/(g dcw × h). In comparison, the hydroxyectoine production with *E. coli* DH5α pASK_ectABCDask under optimal conditions resulted in a specific production rate of 203 mg/(g dcw × h) (Table 2).

**Table 2:** Data on heterologous hydroxyectoine and ectoine production efficiency

	Hydroxyectoine producer (*n* = 9)	Ectoine producer (*n* = 4)
NaCl (%)	0.50	0.25
Growth rate (h^−1^)	0.23 ± 0.02	0.21 ± 0.01
Carbon source (g/L)	4.65	4.65
Dry cell weight (dcw) (g/L)	0.72 ± 0.06	0.57 ± 0.02
Titer (g/L)	1.57 ± 0.13	1.66 ± 0.07
Yield Y_P/S_ (g/g)	0.34	0.36
Yield Y_(X+P)/S_ (g/g)	0.49	0.48
Specific production (g/g dcw)	2.18 ± 0.17	2.93 ± 0.37
Maximum specific production rate^a^ (mg/(g dcw × h))	203 ± 19	345 ± 1

The production values of *E. coli* DH5α pASK_ectABCDask (hydroxyectoine producer) and *E. coli* DH5α pASK_ectABCD_m_ (ectoine producer) are shown for optimum conditions using glycerol as carbon source. Y_P/S_: yield of extracellular hydroxyectoine or ectoine (P) per g substrate (S) consumed, Y_(X+P)/S_: yield of biomass (X) and extracellular hydroxyectoine or ectoine (P) per g substrate (S) consumed

^a^*n* = 2 given as mean with upper/lower boundary

### Carbon balance and maximum yield

As shown in Table 2 our production strains produced considerably more ectoines than biomass, e.g. *E. coli* DH5α pASK_ectABCD_m_ reached almost 3 g of ectoine per g dcw. To elucidate how much carbon source is involved in the heterologous production of ectoines and how much contributes to the formation of biomass, a separated carbon balance was calculated.

Considering the sum of biomass and ectoines as the product, the production strains *E. coli* DH5α pASK_ectABCD_m_ and pASK_ectABCDask reached a yield of 0.48 g and 0.49 g per g glycerol (Y_(X+P)/S_), respectively (Table 2). These values are significantly higher than those of the control strain *E. coli* DH5α with the empty vector (0.38 g/g), generating only biomass under the same conditions. This means carbon is channelled very efficiently into the formation of ectoines in our production strains.

To separate the carbon input for the biosynthesis of ectoines from the biomass formation, the results of the non-producing control strain were used as a reference. Here, the control strain required 2.66 g glycerol (equivalent to 1.04 g carbon) to form 1 g of dry biomass. The hydroxyectoine production strain yielded 0.72 g dry biomass and 1.57 g extracellular hydroxyectoine from 4.65 g glycerol (1.82 g carbon). Since we know from our control strain that 0.75 g carbon are required to generate 0.72 g dry biomass, the remaining 1.07 g carbon were channelled into hydroxyectoine production in *E. coli* DH5α pASK_ectABCDask. From this 0.71 g (66%) carbon could be found directly in the extracellular hydroxyectoine. On a molar basis, this corresponds to a consumption of 30 mmol glycerol for the production of 10 mmol hydroxyectoine. The same ratio applies to the ectoine production strain. Since the carbon of two glycerol molecules is directly channelled into ectoine and hydroxyectoine, respectively, one molecule of glycerol was metabolized otherwise.

The formation of one ectoine molecule (142.2 g/mol) from three glycerol molecules (3 × 92.1 g/mol) results in a yield of 0.51 g/g for the heterologous production. The fact, that this value is fairly close to the combined yield (Y_(X+P)/S_) of 0.48 g/g reflects once more that most of the carbon is channelled into the product during overproduction with our production strain. This yield relates to 62% of the theoretical maximum of 0.77 g/g for a conversion process from two glycerol molecules and no biomass production. Similar results apply to hydroxyectoine (158.2 g/mol) with a value of 57% of the theoretical maximum (0.86 g/g).

### Impact of EctC overexpression on ectoine production

**Fig. 5:**
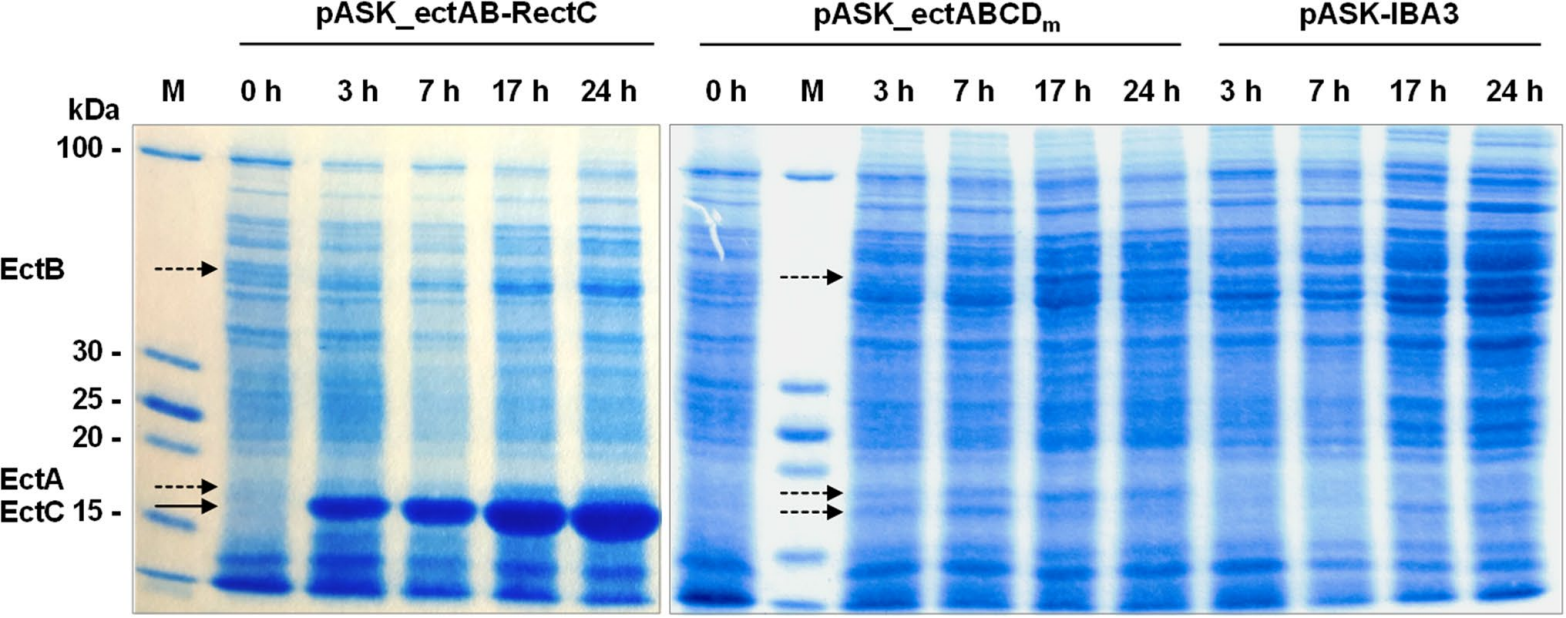
SDS-PAGE of soluble protein extracts purified from ectoine production strains. Soluble proteins were for peer review isolated from *E. coli* DH5α carrying the ectoine producing plasmid pASK_ectABCD_m_, the ectC overexpressing plasmid pASK_ectAB-RectC or the empty vector pASK-IBA3. Samples were taken before induction (0 h) as well as 3, 7, 17, and 24 h after induction. Expected protein bands are marked by arrows. *M* protein marker

As shown in Fig. 3, ectoine overproduction may be hampered by the accumulation of the precursor ADABA, in particular on glucose-containing medium. This points towards a bottleneck for the cyclization reaction catalysed by EctC. In order to improve ectoine production and to reduce extracellular accumulation of the precursor ADABA, we constructed the strain *E. coli* DH5α pASK_ectAB-RectC overexpressing EctC. For this purpose, the plasmid carried the first two genes *ectA* and *ectB* with no alteration to prior constructs, but an additional *tet* promoter and an optimized RBS upstream of *ectC* (Figure S2, Supplementary material). In order to evaluate the protein expression levels of the new plasmid, soluble protein extracts of this strain as well as of the unmodified original ectoine production strain (*E. coli* DH5α pASK_ectABCD_m_) and a control strain with the empty vector were compared by SDS-PAGE (Fig. 5). The successful overexpression of EctC was clearly demonstrated in protein samples of the new strain, while the EctC protein levels were much lower in the case of *E. coli* DH5α pASK_ectABCD_m_.

**Fig. 6:**
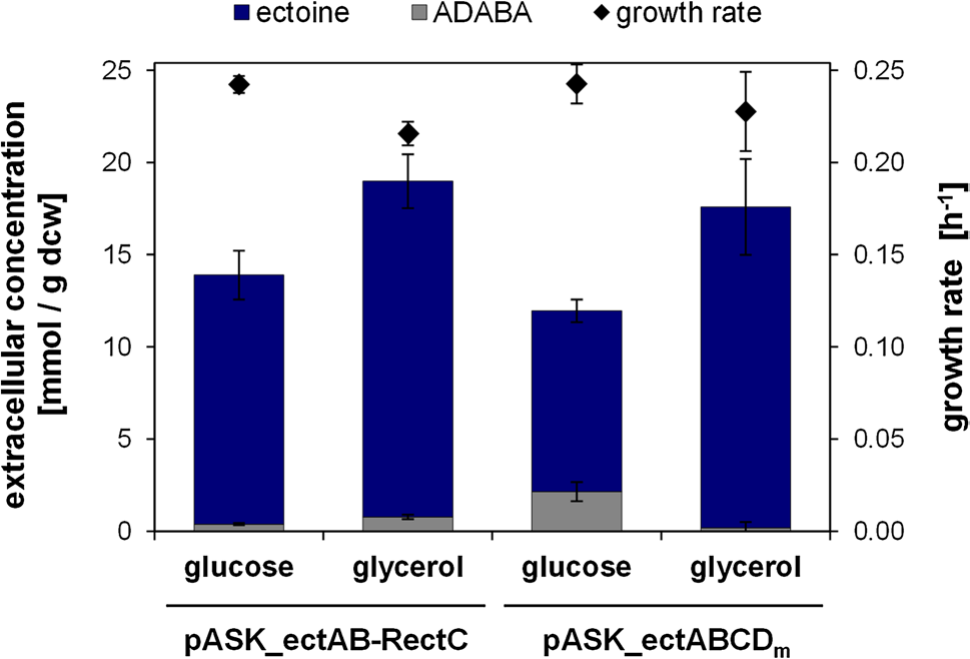
Impact of EctC overexpression on heterologous ectoine production in *E. coli*. The EctC overexpressing strain *E. coli* DH5α pASK_ectAB-RectC and the ectoine production strain *E. coli* DH5α pASK_ectABCDm were cultured in MM63 with 0.5% NaCl and 25 mM glucose or 50 mM glycerol as carbon source. The specific extracellular content of ectoine and its precursor ADABA in mmol/g dry cell weight (dcw), as well as the growth rate (h^–1^), were determined. All experiments were performed at least in triplicates. Error bars indicate the standard deviation (n = 3–20)

Subsequently, shake flask experiments were performed to test the ectoine production capability of the EctC overexpression strain (Fig. 6). Cells were grown in minimal medium with either 25 mM glucose or 50 mM glycerol as carbon source. When utilizing glucose, the specific extracellular ectoine content was higher (13.5 mmol/g dcw vs 9.8 mmol/g dcw) and the ADABA proportion significantly lower (2.8% vs 18%) for the EctC overexpression strain, indicating an optimization in the final step of ectoine synthesis by a raised EctC protein level. Similarly, in glycerol-grown cultures the specific extracellular ectoine content was slightly higher (18.2 mmol/g dcw vs 17.4 mmol/g dcw) for the new strain, but the ADABA share (4.1% vs 0.01%) increased as well (Fig. 6).

## Discussion

### The novel approach of utilizing the non-halophilic hydroxyectoine gene cluster from *A. cryptum* for heterologous overproduction of ectoines

The proteins for hydroxyectoine biosynthesis in *A. cryptum* exhibit a surprisingly low acidity compared to those of halophilic organisms. In halophiles, an increased amount of acidic amino acids (aspartate, glutamate) in the proteome is an adaptation to high cytoplasmic salt concentrations (Oren et al. 2005; Elevi Bardavid and Oren 2012). This adaptation enables correct folding and activity of enzymes under high salt, but reduces their activity under low salt concentrations (Fukuchi et al. 2003). Not surprisingly, therefore only poor activity of the EctC enzyme from the halophilic *H. elongata* (acidity = 10.2%) was recorded under low salt conditions. The activity of the non-halophilic EctC enzyme from *A. cryptum* (acidity = 3%), however, was almost four-times higher at the same low salt conditions (Moritz et al. 2015).

In this study, we were able to confirm the promising attributes of the ectoine/hydroxyectoine biosynthesis proteins from *A. cryptum* for heterologous production processes in *E. coli* under low salt conditions. We here report the highest specific production (g product/g dcw) of ectoines in a heterologous de-novo synthesis system to date. Expression of the gene cluster under control of a suitable promoter for *E. coli* resulted in high extracellular hydroxyectoine and ectoine concentrations (≥ 10 mM) at low salinities (0.25–0.5% NaCl). Interestingly, up to 99% of the ectoines could be found in the culture supernatant without artificial cell wall perturbation, under the low salt conditions employed. Although efflux of osmolytes at low extracellular ion concentration is apparent, the underlying mechanisms remain unclear. *E. coli* exhibits several mechanosensitive channels, which allow a rapid efflux of intracellularly accumulated solutes under hypoosmotic conditions to maintain an osmotic equilibrium (Berrier et al. 1996; Levina et al. 1999). These should, however, not be functional in the absence of hypoosmotic stress. Furthermore, there are indications that mechanosensitive channels are not solely responsible, but specific export systems might be involved in the efflux of compatible solutes in *E. coli* (Czech et al. 2016). In addition, low activity of the osmotically triggered uptake systems ProU and ProP at low salinity might also contribute to the observed accumulation of ectoines in the extracellular space. Although the exact mechanisms of ectoine excretion in *E. coli* remain unclear, the extracellular accumulation greatly facilitates the down-stream product purification and thereby reduces process costs.

### Hydroxyectoine production and influence of aspartokinase from *A. cryptum*

In this study, we were able to record a specific production of hydroxyectoine of up to 2.2 g/g dcw and a maximum specific production rate of 203 mg/(g dcw × h), using the hydroxyectoine gene cluster from *A. cryptum* in a heterologous *E. coli* producer strain. To our knowledge, these are the highest values for heterologous hydroxyectoine production systems reported to date (Table 3). Compared to the current industrial production strain *H. elongata*, which requires NaCl concentrations above 10% to accumulate hydroxyectoine (Wohlfarth et al. 1990), our process with *E. coli* DH5α pASK_ectABCDask required only 0.5% NaCl and could even be performed without the addition of NaCl. Moreover, in our hydroxyectoine production process, only 3.6% of the side product ectoine were present in the supernatant of the production strain. This is in contrast to *H. elongata,* which produces hydroxyectoine only alongside high amounts of ectoine, thus resulting in a time-consuming chromatographic separation of both solutes.

**Table 3:** Comparison of heterologous production strains for ectoine and hydroxyectoine

Production strain	Donor strain	Procedure	NaCl (%)	Titer (g/L)	Specific production (g/g dcw)	Specific production rate (mg/(g dcw × h))	Volumetric productivity^**a**^ (g/(L × h))	Yield (g/g)	Side products^**b**^	References
Ectoine										
* E. coli* DH5α pASK_ectABCD_m_	*A. cryptum*	Batch (flask)	0.25	1.7	2.9	345^c^	–	0.36	Not detected	This study
* E. coli* BL21 pET-ectABC	*H. elongata*	Batch (flask)	0.28	0.8	0.4	~ 200^c^	–	~ 0.09^d^	ADABA	Parwata et al. (2019)
* E. coli* SK51 pLC75	*P. stutzeri*	Batch (flask)	2.3	1.5	1.0	ND	–	~ 0.18^e^	Not indicated	Czech et al. (2018)
* E. coli* ECT05	*H. elongata*	Fed-batch	0	25.1	0.8	~ 125^c^	0.84	0.11^f^	< 7% Ac, 4% α-KG	Ning et al. (2016)
* E. coli* BW25113 pBAD-ectABC	*H. elongata*	Whole-cell catalysis	0	25.1^g^	4.0^g^	~ 250^c,g^	–	ND	Not indicated	He et al. (2015)
* E. coli* DH5α pASK-*ectABC*	*C. salexigens*	Fed-batch	0	6.0	0.3	~ 5^**c**^	0.04	~ 0.02^d^	Not indicated	Schubert et al. (2007)
* C. glutamicum ectABC*^*opt*^	*P. stutzeri*	Fed-batch	0.2	65.3	~ 0.7	120	1.16	0.19	4% Trehalose	Gießelmann et al. (2019)
* C. glutamicum* Ecto15	*C. salexigens*	Fed-batch	0	22.0	~ 1.8	~ 120^**c**^	0.32	0.16	21% Lys, 4% Glu	Pérez-García et al. (2017)
* C. glutamicum* ECT-2^h^	*P. stutzeri*	Fed-batch	0	4.5	ND	ND	0.28	ND	Hydroxyectoine	Becker et al. (2013)
Hydroxyectoine										
* E. coli* DH5α pASK_ectABCDask	*A. cryptum*	Batch (flask)	0.5	1.6	2.2	203^c^	–	0.34	4% ectoine	This study
* E. coli* SK51 pASTI14	*P. stutzeri*	Batch (flask)	2.3	1.9^i^	1.7^i^	ND	–	~ 0.23^i^	~ 32% ectoine	Czech et al. (2018)
				1.3^k^	~ 1.2^k^	ND	–	~ 0.16^e,k^		
* H. polymorpha* ALU3/EctBACD^h^	*H. elongata*	Fed-batch	0	2.8	0.1	~ 2^c^	–	ND	2% ectoine	Eilert et al. (2013)

In all cases, heterologous production and extracellular accumulation of ectoines were achieved under low salt conditions. Values with a tilde ( ~) were calculated from production data given in the references

*α-KG* α-ketoglutarate, *Ac* acetate, *dcw* dry cell weight, *Glu* glutamate, *Lys* lysine, *ND* not determinable

^a^Volumetric productivity corresponds to the ectoine titer in relation to the whole production period

^b^Side products accumulated in the medium in addition to ectoine or hydroxyectoine; the percentage refers to the extracellular total solute content

^c^Maximum specific production rate was calculated from the experimental data and corresponds to the highest increase in extracellular solute concentration relative to the time difference and mean dcw

^d^Yield of extracellular ectoine per g carbon source was calculated from the experimental data

^e^Yield was estimated from the amount of glucose (5 g/L) and aspartate (25 mM = 3.3 g/L) used

^f^Yield refers only to the consumed glucose; the medium additionally contained 2 g/L yeast extract and 4 g/L tryptone of which unknown amounts were consumed

^g^Formation of the biomass used in whole-cell catalysis is not included in the calculation

^h^*ect* genes were codon-optimized and integrated into the genome, while the other heterologous production systems are based on a plasmid

^i^Values refer to hydroxyectoine including the side product ectoine (ca. 32%)

^k^Values refer to hydroxyectoine exclusive the side product ectoine

Interestingly, a direct comparison of hydroxyectoine production with our production strain using either glucose or glycerol as carbon source revealed a higher product formation under glycerol (4.2–8.3 mM vs 5.9–10 mM). This might be explained by the different uptake mechanisms of both carbohydrates in *E. coli*. While glycerol passes the membrane by facilitated diffusion mediated via GlpF (Sanno et al. 1968), glucose is actively transported by the phosphotransferase system using phosphoenolpyruvate (PEP) as phosphoryl donor (Kundig et al. 1964; Mori and Shiio 1985). As a consequence, high glucose consumption leads to high pyruvate and low PEP levels inside the cell. A low PEP-level is disadvantageous for ectoine production by hindering the resupply of the intermediate oxaloacetate, which is drained from the citrate cycle during the production of ectoines (Ning et al. 2016). The accumulation of pyruvate, though, often leads to unwanted side product formation in microbial production processes (Litsanov et al. 2013). Since *E. coli* has no effective measures for converting pyruvate directly to oxaloacetate, the unfavourable pyruvate-PEP levels evoked by glucose consumption might therefore be responsible for the lower product formation under glucose compared to glycerol.

As a possible strategy to improve hydroxyectoine production, we included the aspartokinase of *A. cryptum* into our hydroxyectoine biosynthesis gene cluster for *E. coli*. In 2008, Bestvater et al. had already discovered that the aspartokinase could be a limiting factor for heterologous ectoine production in *E. coli*. Since then, the incorporation of feedback-resistant aspartokinases has become a common strategy to increase product formation of the aspartate amino acid branch and derivatives, e.g. utilizing LysC from *C. glutamicum* (Ning et al. 2016). Some organisms already carry an additional *ask* gene within their ectoine or hydroxyectoine operon, further highlighting its specific role in the production of ectoines. For the *ectABCDask* gene cluster of *Pseudomonas stutzeri* A1501 Stöveken et al. (2011) revealed a positive effect of the additional aspartokinase on the intracellular level of hydroxyectoine when expressed heterologously in *E. coli*. In contrast, Seip et al. (2011) observed no beneficial effect of the aspartokinase using the hydroxyectoine biosynthesis gene cluster from the related *P. stutzeri* strain DSM 5190^T^. In this study, we achieved only a slight improvement in hydroxyectoine production by including the aspartokinase of the *A. cryptum* hydroxyectoine biosynthesis gene cluster. Hence, we hesitate to conclude a beneficial effect of the inherent aspartokinase on the heterologous production of ectoines in *E. coli*. The already very high production rate in this study demonstrates an already efficient process, which might not be significantly limited in aspartokinase activity. Overall, the here described heterologous production of hydroxyectoine at high efficiency and high purity under low salt concentrations could substantially reduce production costs and therefore allow for new applications with this potent protective substance.

### Ectoine production and overexpression of EctC

Ectoine production with the biosynthetic gene cluster of *A. cryptum* led to the highest concentrations of 12 mM in the culture supernatant when grown at 0.25% NaCl. Again, product formation was best when using glycerol as carbon source, leading to a specific production of 2.9 g/g dcw and a maximum specific production rate of 345 mg/(g dcw × h). To our knowledge, these results are unmatched by any other growth-coupled ectoine production process (Table 3). Our protein analysis further demonstrated that a low general level of heterologous proteins is sufficient to generate these high productions. The low EctD protein concentration may also explain the fact that *E. coli* DH5α pASK_ectABCD_m_ showed no significant difference in ectoine production and growth compared to the *E. coli* DH5α strain expressing only the *ectABC* genes.

One of the most critical points for an efficient ectoine production process is the extracellular accumulation of its precursor ADABA, since its uptake is not possible in *E. coli* (Voß 2002). Consequently, the precursor cannot be reintroduced into ectoine biosynthesis after leaving the cell and an elaborate chromatographic separation would be mandatory for high ectoine purities. In our experiments, the ADABA share on the total product was generally negligible in culture media containing glycerol as carbon source and even completely absent at 0.25% NaCl. ADABA formation in glucose cultures, however, was significantly higher and increasing with NaCl concentration. A possible explanation is that high glucose consumption leads to increased growth rates and to overflow metabolism (Holms 1996), negatively affecting EctC expression or activity. By overexpressing EctC we could solve this problem, confirming a bottleneck in EctC activity for our production strain when grown on glucose. Contrary to glucose, we could observe a slight increase in ADABA formation for the EctC overexpression strain when grown on glycerol. The accumulation in very low amounts (< 5%) could yet be inevitable due to a reaction equilibrium of 1:16–20 (≤ 6.25% ADABA) described for EctC of *A. cryptum* in-vitro (Moritz 2019). Since, the overexpression of EctC could successfully reduce the ADABA accumulation for glucose-grown cultures and could increase the overall specific extracellular ectoine concentration slightly under all conditions tested, EctC expression should be considered for further strain optimizations. Including the optimization of EctC expression, we could generate heterologous strains for ectoine production demonstrating unparalleled specific production grown on glycerol (2.9 g/g dcw) or glucose (2 g/g dcw).

### Carbon and energy balance

**Fig. 7:**
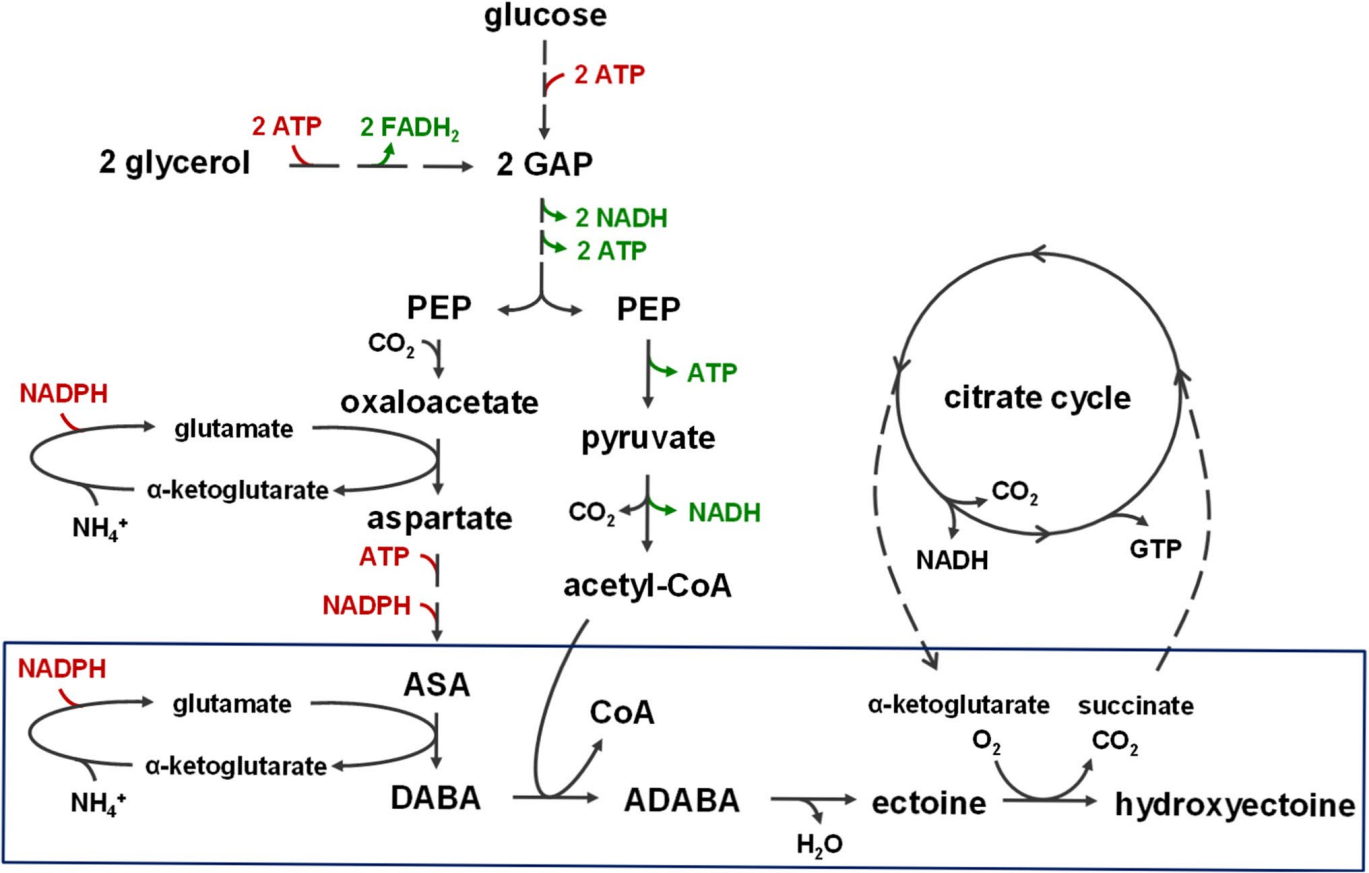
Energy and co-factor balance of heterologous ectoine and hydroxyectoine biosynthesis in *E. coli*. The diagram shows the main intermediates of the ectoine and hydroxyectoine formation from glycerol and glucose, respectively, as well as co-factors used and generated on route. The reactions of the heterologous biosynthetic pathway are indicated by a blue box. Ectoine biosynthesis needs one molecule of L aspartate-βsemialdehyde and acetyl-CoA. In the best-case scenario, oxaloacetate is directly supplied by carboxylation of PEP, while acetyl-CoA is generated from a second PEP. Theoretically, one molecule of glucose or two molecules of glycerol could be sufficient for the generation of one molecule of ectoine. If, however, oxaloacetate is replenished via the glyoxylate cycle, three rather than two molecules of glycerol would be required. In the case of hydroxyectoine production the consumption of α-ketoglutarate leads to a shortcut of the citrate cycle, resulting in the loss of 1 NADH and 1 GTP for every turn of the cycle. *ASA* L-aspartate-βsemialdehyde, *CoA* Coenzyme A, *GAP* glyceraldehyde 3-phosphate, *PEP* phosphoenolpyruvate

In addition to the optimal energy and carbon balance for ectoine biosynthesis with glucose or glycerol in *E. coli* as depicted in Fig. 7, two molecules of glutamate are needed to supply the amino groups. Assuming that the glutamate dehydrogenase is active in *E. coli* DH5α under the given growth conditions, one NADPH molecule is required for the regeneration of one glutamate from α-ketoglutarate. Following the best-case pathway with glucose as substrate, ATP consumption and production would balance out, while three molecules of NADH are generated and three molecules of NADPH are consumed in biosynthesis. The regeneration of NADPH by NADH, however, requires the input of ATP. When using the more reduced substrate glycerol, two additional molecules of FADH_2_ are generated, resulting in up to four additional molecules of ATP, which could drive NADPH regeneration.

The carbon balance of our ectoine and hydroxyectoine producer strains revealed that an additional glycerol molecule is consumed during heterologous production in *E. coli*. Possible reasons are the need for NADPH regeneration, partial use of the glyoxylate cycle for oxaloacetate synthesis, the biosynthesis of the recombinant proteins and maintenance energy needed for a system stressed by overproduction and excretion of products two to three times the amount of its cell mass. Compared to the best-case scenario, the substrate glycerol is very efficiently channelled into ectoine production in the strain we generated.

### Comparison of production strains for ectoine and hydroxyectoine

At present, industrial production of ectoines is primarily performed with the halophilic natural-producer strain *H. elongata*. The production process is facilitated by using ectoine-excreting mutants, so-called leaky mutants (Kunte et al. 2014). Extracellular accumulation of ectoines has the advantages that the cell density does not limit the product titer and that downstream processes can be simplified. Nevertheless, the production of ectoines using *H. elongata* depends on the addition of significant amounts of NaCl, still reducing product revenue. In the case of hydroxyectoine, high amounts of its precursor ectoine are accumulated aside the main product in *H. elongata*, demanding even more elaborate downstream processes. By now, however, already a few heterologous production strains have been developed that achieve accumulation of ectoines in the medium at low salinity or even without the addition of NaCl. For an appropriate comparison with our production strains, we determined the specific production of ectoines, the maximum specific production rate and the yield of ectoines based on the total amount of carbon source consumed, additionally to the product titer (Table 3).

He et al. (2015) performed a whole-cell biocatalysis of ectoine from aspartate and glycerol using *E. coli* with the *ectABC* gene cluster of *H. elongata*, which yielded a very high specific ectoine production of 4.1 g/g dcw. However, since this is the only whole-cell biocatalysis process described, these results are not directly comparable with the specific production of the other de-novo production systems. The formation of the starting biomass used in this whole-cell biocatalysis has not been included in the calculation, leading to the high specific production values. Furthermore, in order to produce 4.1 g/g dcw of ectoine more than 0.35 M of the expensive precursor aspartate (> 46 g/L) were consumed, in addition to 0.2 M glycerol (> 18 g/L). This equals a low substrate yield of the process.

Schubert et al. (2007) described the first heterologous ectoine production and excretion by *E. coli* using a culture medium without the addition of NaCl. Despite using a very similar host system (*E. coli* DH5α and pASK-IBA7) a significantly lower specific production and maximum specific production rate were reached, compared to our strain *E. coli* DH5α pASK_ectABCD_m_. This might be connected to the *ectABC* gene cluster from the halophilic bacterium *Chromohalobacter salexigens* Schubert et al. utilized.

Gießelmann et al. (2019) generated a *C. glutamicum* strain with the ectoine biosynthesis genes from *P. stutzeri* using a monocistronic approach with balanced transcription of the individual ectoine synthesis genes. The optimal relative expression of the heterologous enzymes was analysed by generating and screening a transcription library. In a fed-batch fermentation with sugar and molasses as carbon sources, the production strain achieved a remarkable ectoine titer of 65.3 g/L, which is the highest value reported so far. The authors also described a promising specific production rate of 120 mg/(g dcw × h). These data show that a halophilic gene cluster can work well in the Gram-positive organism *C. glutamicum*.

A high ectoine titer of up to 25.1 g/L was obtained by Ning et al. (2016) with an *E. coli* strain carrying the *ectABC* operon from *H. elongata* 1A01717 under control of the IPTG-inducible *trc* promoter. The productivity was increased through several metabolic engineering steps, e.g. overproduction of the PEP carboxylase reinforcing the oxaloacetate pool, and expression of a feedback-resistant aspartokinase from *C. glutamicum*. Overall metabolic engineering resulted in a good maximum ectoine production rate of approx. 125 mg/(g dcw × h) during fed-batch fermentation with complex medium and glucose as the main carbon source.

Compared with the (de-novo) production systems described so far in the literature, our ectoine production strain achieves the highest specific production, production rate and yield, but is limited in product titer. For industrial relevance, the novel and unique potentials of the non-halophilic gene cluster from *A. cryptum* need to be transferrable into upscaled bioreactor processes.

For hydroxyectoine production only two other heterologous production systems have been described to date, which enable an extracellular hydroxyectoine accumulation at low NaCl concentrations (Table 3).

Eilert et al. (2013) utilized the yeast *Hansenula polymorpha* as host for heterologous hydroxyectoine production. The genes *ectABCD* from *H. elongata* were codon-optimized and integrated into the genome of *H. polymorpha*. During fed-batch fermentation with methanol, glycerol, and sorbitol as carbon sources, a hydroxyectoine titer of 2.8 g/L could be reached with a low ectoine share of 2%. However, the specific hydroxyectoine production and maximum specific production rate were very low when compared to *E. coli* DH5α pASK_ectABCDask.

Czech et al. (2018) generated an *E. coli* strain extracellularly accumulating hydroxyectoine as the main product. The highest specific hydroxyectoine production of approx. 1.2 g/g dcw was achieved after several genetic modification steps and by using a culture medium with 2.3% NaCl and glucose as well as aspartate as substrate. Under the optimal conditions, a mixture of hydroxyectoine and ectoine with a precursor share of 32% was accumulated in the culture medium (1.9 g/L total ectoines). In contrast, our hydroxyectoine-excreting *E. coli* strain reached almost twice the specific hydroxyectoine production with a lower share of the precursor ectoine (4%).

In this manuscript we are the first to report an ectoine and hydroxyectoine production based on the biosynthesis gene cluster of a non-halophilic bacterium in *E. coli*. The strains generated achieve unsurpassed specific production and production rates at low NaCl concentrations and from cheap carbon sources. We believe that the first-ever use of a non-halophilic gene cluster for heterologous overproduction of ectoines may be accountable for this success and should be of pronounced interest for efficient heterologous production of ectoines in *E. coli*. A more detailed investigation of the performance differences of halophilic and non-halophilic gene cluster for heterologous production would be of interest for upcoming studies. The product titers and cell densities in the here presented shake flask experiments are at a basal level and should be upscaled in the next steps. The data presented, however, already demonstrate an impressive potential of the employed non-halophilic gene cluster in *E. coli*. For an industrial application additional improvement is necessary, e.g. the use of a suitable *E. coli* base-strain for industrial production, as well as metabolic engineering and optimization of process conditions. Until this point however, this novel attempt for the heterologous production of ectoines at low NaCl concentration was rewarded with a benchmark for specific production and production rates. Furthermore, the produced ectoines are mainly accumulated in the medium (≥ 99%), and the proportion of unwanted side products is minimal (≤ 4%). Because of these benefits, the use of the non-halophilic gene cluster presented in this study could open new opportunities for the industrial production and purification process of ectoines.

## Electronic supplementary material

Below is the link to the electronic supplementary material.

Supplementary file1 (DOCX 960 kb)
