# Supplementary material for: Boosting Escherichia coli’s heterologous production rate of ectoines by exploiting the non-halophilic gene cluster from Acidiphilium cryptum
Source: Extremophiles. 2020 Jul 22;24(5):733–47. doi: 10.1007/s00792-020-01188-8 (PMC7445199; doi:10.1007/s00792-020-01188-8)
Supplement: Supplementary file 1 — Supplementary file1 (DOCX 960 kb) [file 792_2020_1188_MOESM1_ESM.pdf]

Supplementary Material

Boosting *Escherichia coli´s* heterologous production rate of ectoines by exploiting the non-halophilic gene cluster from *Acidiphilium cryptum*

Extremophiles

Lukas Bethlehem[1+*] and Katharina D. Moritz[1+]

[1]Institute for Microbiology & Biotechnology, University Bonn, Meckenheimer Allee 168, 53115 Bonn, Germany

[+]Equal contributors

[*]Correspondence: ifmb@uni-bonn.de

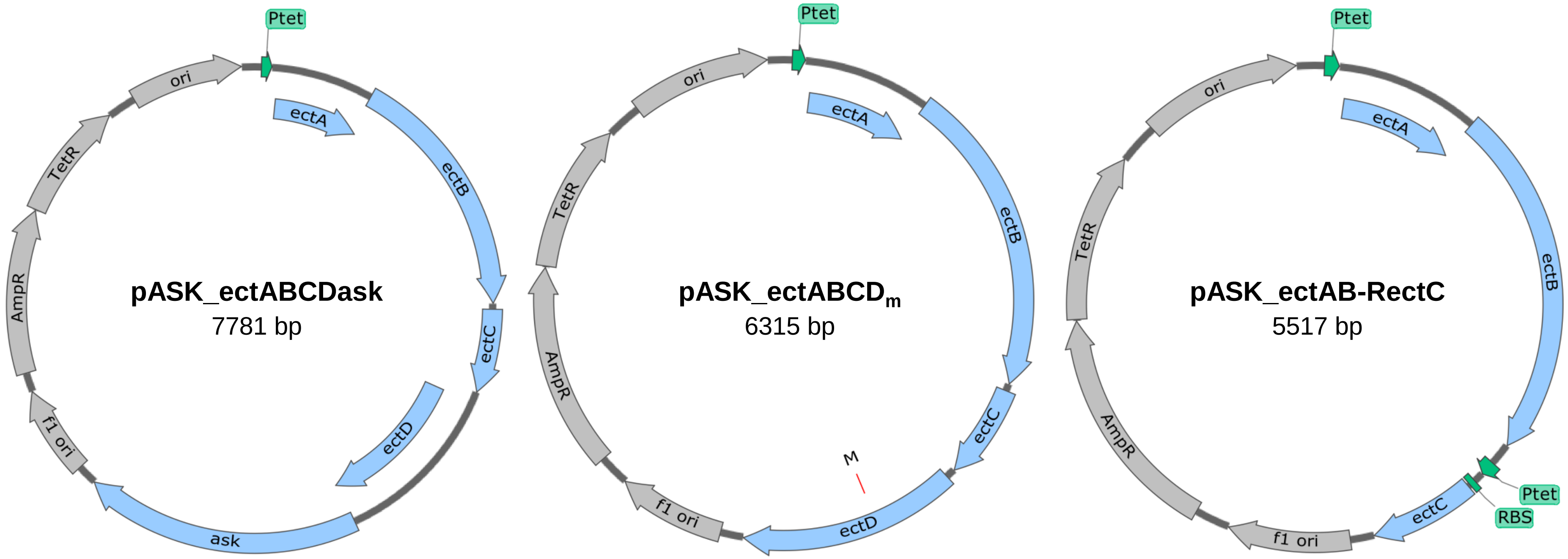

Fig. S1 Plasmids for overproduction of ectoines in *E. coli* utilizing the gene cluster from *A. cryptum*. The plasmid pASK_ectABCDask was used for heterologous overproduction of hydroxyectoine, and the plasmid pASK_ectABCD$_m$ containing a random mutation in *ectD* (M) was employed for heterologous ectoine overproduction. The plasmid pASK_ectAB-RectC overexpressing *ectC* under the control of a separate *tet* promoter (Ptet) and the optimized ribosome binding site (RBS) from the pET-22b(+) vector was constructed to minimize precursor production. AmpR: ampicillin resistance, ori: origin of replication, TetR: *tet* repressor

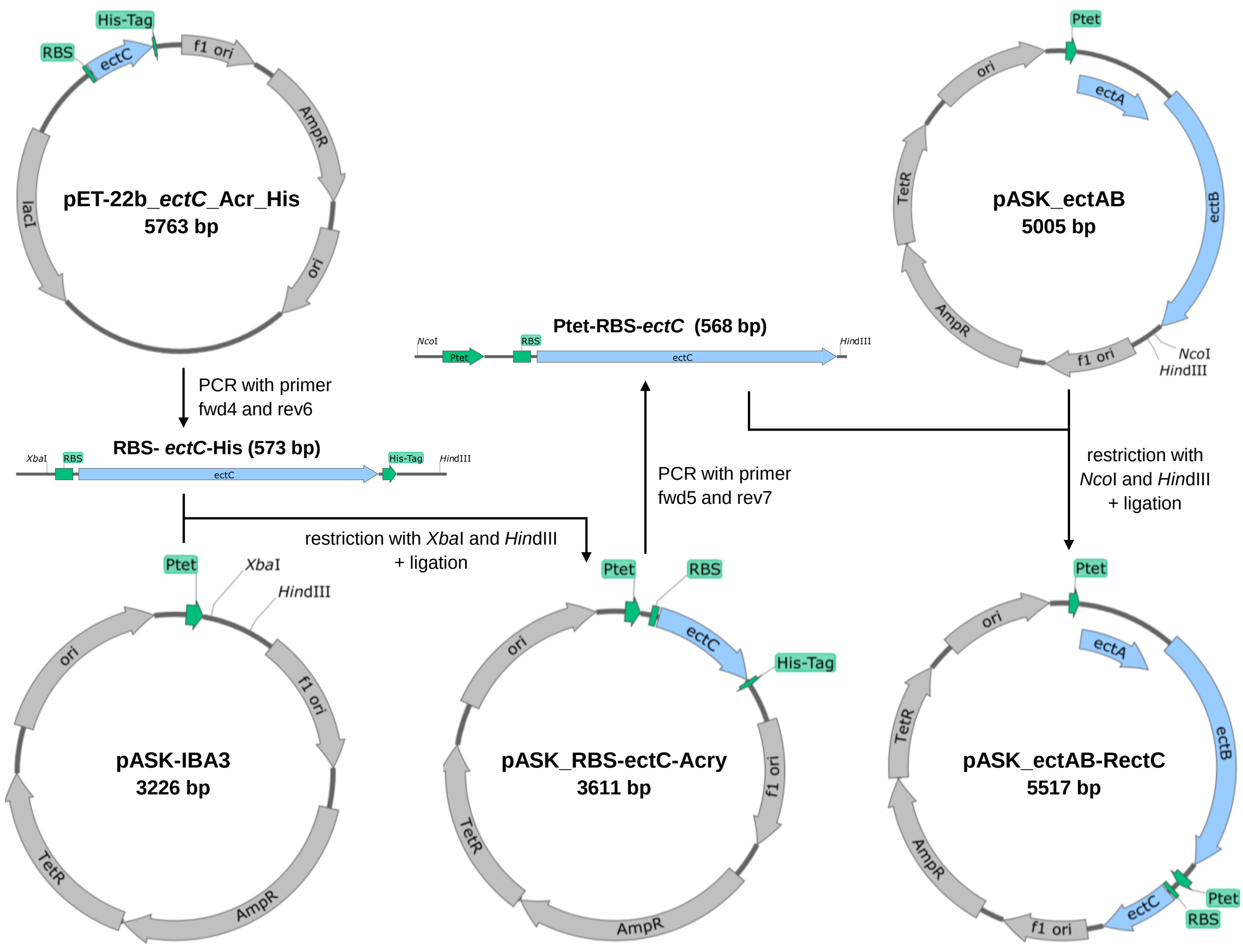

Fig. S2 Construction of plasmid pASK_ectAB-RectC. The *ectC* gene of *A. cryptum* was amplified together with the optimized ribosome binding site (RBS) of the pET-22b(+) vector using plasmid pET-22b_*ectC*_Acr_His as the template. For construction of pET-22b_*ectC*_Acr_His, *ectC* was amplified from the *A. cryptum* genome with the primers fwd3 and rev5, and integrated into pET-22b(+) after restriction with *Nde*I and *Xho*I (Moritz et al. 2015). After integration of the *ectC* construct into the pASK-IBA3 vector to form pASK_RBS-ectC-Acry, another PCR was performed to obtain the construct with the *ectC* gene under control of the *tet* promoter (Ptet) and the optimized RBS from pET-22b(+). In another cloning step, the PCR product was integrated into pASK_ectAB containing the genes *ectA* and *ectB* downstream of a *tet* promoter to generate the plasmid pASK_ectAB-RectC. For construction of pASK_ectAB, the genes *ectA* and *ectB* were amplified from the *A. cryptum* genome using the primers fwd1 and rev8, and integrated into pASK-IBA3 after restriction with *Xba*I and *Nco*I. The sequences of the primers used are given in Table 1 in the material and methods section. AmpR: ampicillin resistance, lacI: *lac* repressor, ori: origin of replication, TetR: *tet* repressor

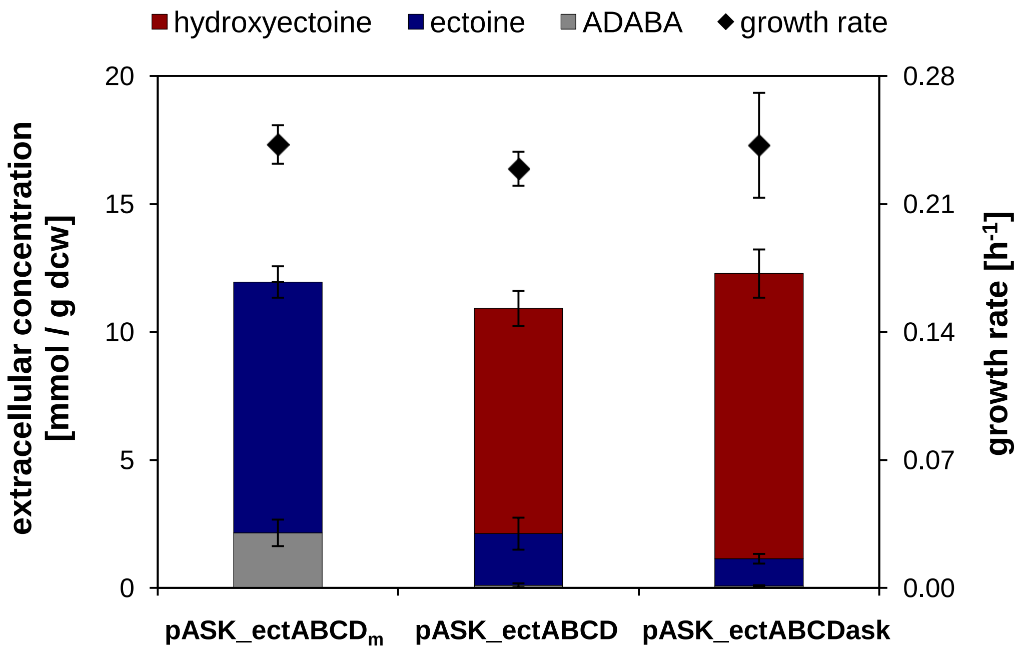

Fig. S3 Comparison of product formation using different gene cluster compositions. *E. coli* DH5α carrying the plasmid pASK_ectABCD$_m$ (mutation in *ectD*), pASK_ectABCD or pASK_ectABCDask was cultured in MM63 supplemented with 0.5 % NaCl and 25 mM glucose. The specific extracellular concentration of hydroxyectoine, ectoine, and its precursor ADABA in mmol/g dry cell weight (dcw), as well as the growth rate (h^{-1}), were determined. All experiments were performed at least in triplicates. Error bars indicate the standard deviation (n=3-10).

Moritz KD, Amendt B, Witt EMHJ, Galinski EA (2015) The hydroxyectoine gene cluster of the non-halophilic acidophile *Acidiphilium cryptum*. Extremophiles 19:87-99
